# Comparison of prophylactic effects for chemotherapy induced neutropenia between same-day versus next-day administration of pegteograstim (Neurapeg®) in patients treated with chemotherapy regimen composed of day 1 intensive myleosuppressive agent: A randomized phase III clinical trial

**DOI:** 10.1097/MD.0000000000033638

**Published:** 2023-05-17

**Authors:** Kwonoh Park, Young-Kyung Jeon, Jung Hoon Kim, Younak Choi, Jae-Joon Kim, Sang-Bo Oh, So Yeon Oh, Yun Jeong Hong, Seok Jae Huh, Ilhwan Kim, Seong Hoon Shin

**Affiliations:** a Medical Oncology and Hematology, Department of Internal Medicine, Pusan National University Yangsan Hospital, Pusan National University School of Medicine, Yangsan, Korea; b Department of Internal Medicine, Hanyang University Seoul Hospital, Hanyang University College of Medicine, Seoul, Korea; c Department of Neurology, Uijeongbu St. Mary’s Hospital, Catholic University of Korea, Seoul, Korea; d Department of Internal Medicine, Dong-A University College of Medicine, Busan, Korea; e Division of Oncology, Department of Internal Medicine, Haeundae Paik Hospital, Inje University College of Medicine, Busan, South Korea; f Department of Internal Medicine, Kosin University Gospel Hospital, Kosin University College of Medicine, Busan, Korea.

**Keywords:** chemotherapy-induced neutropenia, pegteograstim, pegylated GCSF, prophylaxis

## Abstract

Administration of pegylated granulocyte-colony-stimulating factor (peg-GCSF) 24 to 72 hours after chemotherapy is usually recommended. Next-day administration (after 24 hours) resulted in fewer duration of grade (Gr) 4 chemotherapy-induced neutropenia (CIN) and decreased severity of CIN than same-day (within 4 hours). However, patients sometimes receive same-day Peg-GCSF for the sake of convenience. In addition, a few prior studies showed that the same-day method is comparable or superior to the next-day method in preventing CIN, especially in chemotherapy regimens that include day 1 myelosuppressive agents. Thus, we aim to verify the hypothesis that same-day administration of pegteograstim, a new formulation of peg-GCSF, is non-inferior to next-day administration in terms of Gr4 CIN duration.

**Methods:**

This study is a randomized, multicenter, open-label, investigator-initiated phase 3 study. Patients with adjuvant/neoadjuvant or first-line palliative chemotherapy comprising intensively myelosuppressive agents on day 1 (mFOLFIRINOX, ECb, EP, FOLFIRI, and FOLFOX) are enrolled. The patients are assigned to the same-day arm or the next-day arm in a 1:1 ratio. The randomizations are stratified according to number of patient CIN risk factors (1 vs ≥2), chemotherapy setting (perioperative vs palliative), and interval (2-week vs 3-week). In the same-day arm, pegteograstim 6 mg is subcutaneously injected within 4 hours after completion of chemotherapy. In the next-day arm, pegetograstim is injected at 24 to 36 hours post-chemotherapy. A complete blood count test is performed daily from day 5 to 9 during the cycle 1. The primary endpoint is duration of Gr4 CIN (cycle 1), and secondary endpoints include incidence of Gr 3 to 4 CIN (cycle 1), severity of CIN (cycle 1), time to recovery absolute neutrophil count 1000/μL (cycle 1), incidence of febrile neutropenia, incidence of CIN-related dose delay, and dose intensity. In order to verify non-inferiority of 0.6 days, we estimated a significance level of 5%, power of 80%, and drop-out rate of 15%. This results in the need for a total of 160 patients, 80 in each group.

## 1. Introduction

### 1.1. Background

Chemotherapy-induced neutropenia (CIN) is one of representative adverse events (AEs) of cytotoxic chemotherapy that^[1]^ increases risk of febrile neutropenia (FN) and related mortality,^[2]^ reduces the effects of chemotherapy due to dose-limiting toxicity, and^[3]^ increases costs related to treatment of FN.^[4]^ Since CIN occurs most frequently during the first cycle of chemotherapy and adversely affects the clinical outcome, primary prophylaxis using granulocyte-colony-stimulating factor (GCSF) is recommended at the beginning of chemotherapy cycle 1 in patients with high-risk or moderate risk with risk factors.^[1]^

Pegylated granulocyte-colony-stimulating factor (peg-GCSF) was developed to increase the half-life to 46 to 72 hours by attaching polyethylene glycol to filgrastim so that only 1 administration per cycle is necessary to prevent CIN. A single injection of peg-GCSF showed an equivalent effect to daily filgrastim therapy in a comparative study.^[5]^ Based on the mechanism of action of peg-GCSF and results of prior prospective and meta-analysis studies,^[3,6]^ administration of peg-GCSF 24 to 72 hours after completion of chemotherapy is recommended.^[1]^ Patients in which the next-day method (administration after 24 hours post-chemotherapy) was implemented had fewer days in Grade (Gr) 4 neutropenia than the same-day method (administration within 4 hours post-chemotherapy). CIN was also less severe in the next-day group.

However, approximately 13% of patients receive same-day peg-GCSF probably due to the inconvenience of providing an additional hospitalization or hospital visit for administration of the peg-GCSF.^[7]^ In addition, in some retrospective studies or subgroup analyses, the same-day method was comparable or superior to the next-day method in preventing CIN in multi-day (3 to 5 days) regimens in which myelosuppressive agent administration was concentrated on day 1.^[3,8]^ Therefore, there is a need to reevaluate the efficacy of the same-day method in such cases.

Pegteograstim is a new formulation of peg-GCSF, that was developed by Green Cross Corporation in Korea. Pegteograstim was compared with the pegfilgrastim in a 2016 phase II/III study. There was no significant difference in Gr4 CIN duration (pegteograstim 1.97 days, pegfilgrastim 1.54 days, *P* = .33), and pegteograstim showed a significantly shorter CIN recovery time (pegteograstim 8.85 days, pegfilgrastim 9.83 days, *P* < .001).^[9]^

### 1.2. Study aims

Our study aim is to test the hypothesis that same-day administration of pegteograstim is non-inferior to next-day administration in controlling CIN Gr4 duration during first cycle in patients treated with chemotherapy regimens that contain intensively myelosuppressive agents on day 1.

## 2. Methods

### 2.1. Study design

**Figure 1.**
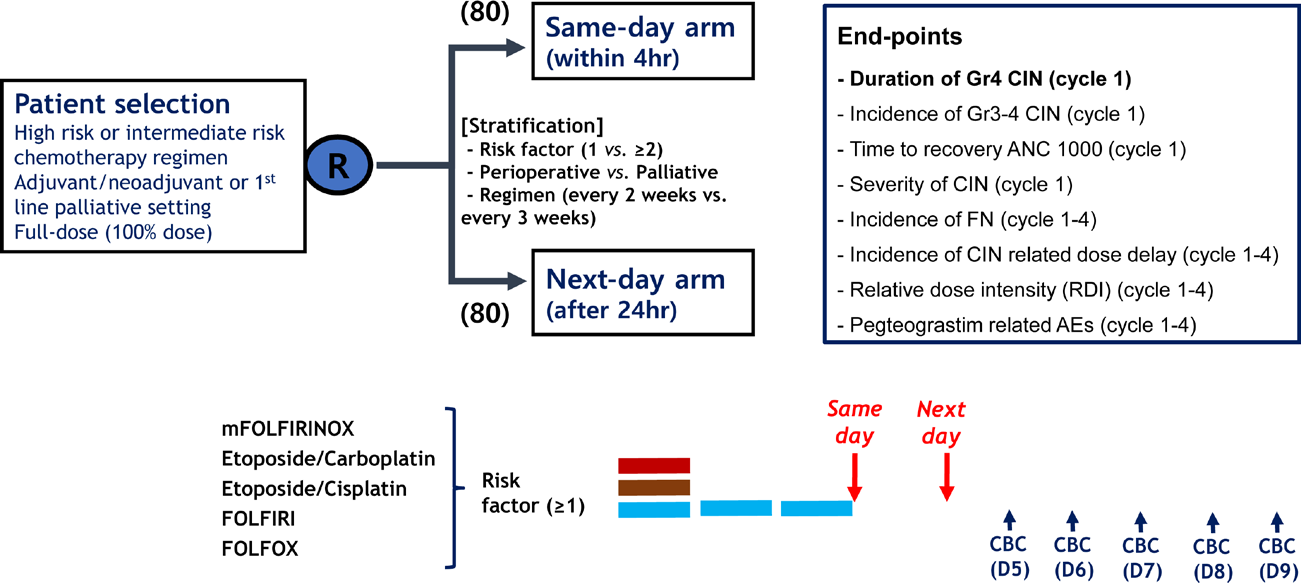
Study flowchart.

This study is a randomized, multicenter, parallel, open-label, phase III study. This study is designed to test the non-inferiority of same-day to next-day administration of pegteograstim (Fig. 1).

### 2.2. Study registration

This study trial has been registered on the Clinical Research Information Service (http://cris.nih.go.kr) with the ID No. KCT0007694 (Release date: September 15, 2022).

### 2.3. Participants

Patients with adjuvant/neoadjuvant or first-line palliative chemotherapy with regimen containing an intensively myleosuppressive agent on day 1 are enrolled.

Enrollment criteria are: Patients with histologically confirmed malignancy who are scheduled for adjuvant/neoadjuvnat or first-line palliative chemotherapy with multi-day combination regimens. These regimens include mFOLFIRINOX for Pancreatic cancer, ECb and EP for Small cell carcinoma, Neuroendocrine carcinoma, and FOLFOX and FOLFIRI for colorectal cancer. Detailed contents of these regimen are described in Table 1. The patients receiving FOLFOX and FOLFIRI combination therapies with targeted agents such as cetuximab or bevacizumab are also included, and those receiving atezolizumab in addition to the ECb and EP regimens for small cell lung carcinoma are also eligible; Patients with 1 or more risk factors for CIN: 65 years of age or older, previous history of chemotherapy or radiation therapy, recent surgery and open wounds, liver dysfunction with total bilirubin > 2.0 mg/dL, renal dysfunction with creatinine clearance < 50 mL/minutes. An exception is that patients on the mFOLFIRINOX regimen can be enrolled without CIN risk factors due to the high-risk for CIN of this regimen; Patients who are scheduled to receive 100% dose of chemotherapy; Patients between the ages of 19 and 75; Patients with Eastern Cooperative Oncology Group performance status 0 to 1; Patients with adequate bone marrow function for chemotherapy [absolute neutrophil count (ANC) ≥ 1500/μL], (platelets ≥ 100,000/μL, and hemoglobin ≥ 9.0 g/L), adequate renal function (creatinine clearance ≥ 40 mL/minutes), adequate liver function [total bilirubin in the blood < 1.5 × upper limit normal (ULN), alanine aminotransferase or aspartate aminotransferase  < 3.0 × ULN. The alanine aminotransferase or aspartate aminotransferase value must be ≤ 5.0 × ULN in patients with documented disease metastases to the liver); Patients who agreed to participate in the study.

**Table 1:** Chemotherapy regimens.

Cancer type	Regimen		Day
Pancreatic cancer	mFOLFIRINOX		Every 2 wk
	5-FU	1200 mg/m^2^ IV continuous infusion	d 1–2
	Oxaliplatin	85 mg/m^2^ IV	d 1
	Irinotecan	150 mg/m2 IV	d 1
Small cell carcinoma, Neuroendocrine carcinoma	EP*		Every 3 wk
	Etoposide	100 mg/m^2^ IV	d 1–3
	Cisplatin	70 mg/m^2^ IV	d 1
Small cell carcinoma, neuroendocrine carcinoma	ECb*		Every 3 wk
	Etoposide	100 mg/m^2^ IV	d 1–3
	Carboplatin	AUC 6.0 mg/mL IV	d 1
Colorectal cancer	FOLFOX†		Every 2 wk
	5-FU	3000 mg/m^2^ IV continuous infusion	d 1–2
	Oxaliplatin	85 mg/m^2^ IV	d 1
	Leucovorin	400 mg/m2 IV	d 1
Colorectal cancer	FOLFIRI†		Every 2 wk
	5-FU	3000 mg/m^2^ IV continuous infusion	d 1–3
	Leucovorin	400 mg/m2 IV	d 1
	Irinotecan	180 mg/m2 d 1	d 1

* Combination therapies with atezolizumab are also included.

† Combination therapies with targeted agents such as cetuximab or bevacizumab are also included.

The exclusion criteria are: Patients aged 76 years or older, Patients unable to receive 100% dose intensity in cycle 1; Patients who underwent radiation therapy within 1 month before starting treatment; Patient with other serious diseases or medical conditions; Pregnant or lactating women or those of childbearing age who do not consider contraception.

### 2.4. Recruitment

We will recruit 160 participants scheduled to receive multi-agent chemotherapy regimens with intensively myleosuppressive agents on day 1 (mFOLFIRINOX, ECb, EP, FOLFOX, FOLFIRI) as an adjuvant/neoadjuvant or first-line palliative treatment in 5 Korean academic hospitals (Pusan national university yangsan hospital, Hanyang University Seoul Hospital, Dong-A University hospital, Haeundae Paik Hospital, Kosin University Gospel Hospital) (Fig. 1). The researcher explains the aim and the methods of the study to the participants and obtains informed consent from potential participants before the collection of medical information.

### 2.5. Intervention

For those in the same-day arm, pegteograstim 6mg is subcutaneously injected within 4 hours after completion of chemotherapy. Those in the next-day arm receive pegteograstim 6mg subcutaneously 24 to 36 hours after completion of chemotherapy.

### 2.6. Randomization and stratification

The patients are randomized 1:1 in a centralized block randomization manner to be allocated to either the same-day or the next-day arm. The randomization is stratified according to number of patient’s CIN risk factors (1 vs ≥ 2), chemotherapy setting (perioperative vs palliative), and interval of regimen (2-week vs 3-week). This clinical study is an open-label trial; both the investigator and the patient know which method is being administered.

### 2.7. Evaluation and outcome measure

During cycle 1, a complete blood count (CBC) test is performed every day from day 5 to 9. If the ANC rises for 2 consecutive days before day 9 and is over 1000/mm^3^, CBCs can be stopped. Conversely, if the ANC does not recover to 1000/mm^3^ or more until day 9, the daily CBC should be continued until the ANC recovers to 1000/mm^3^. From cycle 2 to 4, anticancer treatment is performed according to general practice; and neutropenic fever, dose of chemotherapy, and AEs are assessed.

### 2.8. Outcome measures

Outcome measures are listed in Table 2.

**Table 2:** Detailed descriptions of the endpoints.

Endpoints	Explanation
Duration of Gr4 CIN (cycle 1)	Duration (in d) of absolute neutrophil count (ANC) < 500/μL in the first chemotherapy cycle
Incidence of Gr 3–4 CIN (cycle 1)	Subjects with ANC < 1000/μL in the first chemotherapy cycle
Severity of CIN (cycle 1)	Minimum ANC level in the first chemotherapy cycle
Time to ANC recovery (cycle 1)	Time (in d) from day 1 of chemotherapy to ANC > 1000/μL after the nadir in the first chemotherapy cycle
Febrile neutropenia (FN) incidence (cycle 1–4)	Development of FN until 28 d after the end of cycle 4. FN is defined as an oral temperature rise to 38.3°C or higher or a fever of 38.0°C or higher that lasts for 1 h or more with an ANC <500/mm^3^ or with an ANC <1000/mm^3^ that is expected to decrease to <500/mm^3^ within 48 h
Incidence of CIN-related dose delay (cycle 1–4)	Delay of chemotherapy schedule due to development of CIN
Relative dose intensity (RDI) (cycle 1–4)	Dividing the total dose of chemotherapy (mg/m^2^) by the total administration period, and the value was expressed as a percentage based on the full dose intensity (Table 1)
Pegteograstim-related AEs (cycle 1–4)	Occurrence of bone pain and pneumonitis during cycles 1–4

AEs = adverse events, CIN = chemotherapy induced neutropenia, Gr = grade.

- Duration of Gr4 CIN (cycle 1): duration (in days) of ANC < 500/μL in the first chemotherapy cycle.
- Incidence of Gr 3 to 4 CIN (cycle 1): subjects with ANC < 1000/μL in the first chemotherapy cycle.
- Severity of CIN (cycle 1): minimum ANC level in the first chemotherapy cycle.
- Time to ANC recovery (cycle 1): time (in days) from day 1 of chemotherapy to ANC > 1000/μL after the nadir in the first chemotherapy cycle.
- FN incidence (cycle 1–4): development of FN until 28 days after the end of cycle 4. FN is defined as an oral temperature rise to 38.3°C or higher or a fever of 38.0°C or higher that lasts for 1 hour or more with an ANC <500/mm^3^ or with an ANC <1000/mm^3^ that is expected to decrease to <500/mm^3^ within 48 hours.
- Incidence of CIN-related dose delay (cycle 1–4).
- Relative dose intensity (RDI) (cycle 1–4): RDI is calculated by dividing the total dose of chemotherapy (mg/m^2^) by the total administration period, and the value was expressed as a percentage based on the full dose intensity (Table 1).
- Pegteograstim-related AEs (cycle 1–4): the occurrence of bone pain and pneumonitis during cycles 1 to 4.

### 2.9. Criteria for discontinuation of study drug and study drop-out

Criteria for discontinuation of the study drug are: Completion of the planned 4 cycles; Disease progression according to RECIST v1.1, or unacceptable AEs requiring discontinuation of chemotherapy; Withdrawal of consent, and pregnancy.

The drop-out criteria are; withdrawal of consent and; pregnancy.

### 2.10. Sample size calculation

The primary endpoint of the study is duration of Gr4 CIN. In prior studies using the next-day administration method, the duration of Gr4 CIN was approximately 2 to 2.5 days. Considering the non-inferiority margin of 0.6 days and equivalence margins of 1 to 2.1 days in previous studies,^[2,3]^ 0.6 days of Gr4 CIN duration was selected for the non-inferiority margin. In order to verify the non-inferiority of 0.6 days (standard deviation 1.4 days), a significance level of 5% and power of 80% were estimated. This resulted in the need for 136 patients, 68 per group. Adjusting for sample size by expecting a drop-out rate of 15% in each treatment group, we planned for enrollment of 160 total subjects, 80 subjects in each treatment group. The study subject registration period was assumed to be 2 years, and the follow-up period was assumed to be 3 months.

### 2.11. Statistical analysis

The primary analysis is performed in the per-protocol (PP) set, considering the non-inferiority design. The per-protocol group is defined as patients who do not have any of these following significant deviations from the clinical trial protocol: Failure to administer the study drug (pegteograstim) within the scheduled period (same-day arm: within 4 hours, Next-day arm: 24–36 hours); Discontinuation of CBCs before < 50% of tests (on 3 or fewer days) or until ANC reaches > 500/μL, and; development of FN and treatment with additional filgrastim at the discretion of the investigator.

Demographics, clinical presentation, perioperative clinical findings, and laboratory values are summarized using descriptive statistics. Continuous variables are described by median values, and categorical variables are described by absolute numbers and percentages. *T* test is used for group comparison of continuous variables such as duration of Gr4 CIN, severity of CIN, and RDI. Chi-square test or Fisher exact test is used for group comparison of categorical variable values such as incidence of Gr 3 to 4 CIN (Cycle 1), FN, CIN-related dose delay, and pegteograstim-related AEs. Time to ANC recovery is analyzed by the method of Kaplan–Meier.

### 2.12. Data collection and study ethics

Data will be de-identified and recorded on electronic case report forms. Access to the electronic case report forms links will be restricted to the study investigators. If a subject withdraws from the study, reasons for the withdrawal will be recorded and all attempts will be made to collect endpoint data. Access to the participant’s personal information will be restricted to the study coordinator and investigators who are involved in the screening.

This investigator-initiated clinical trial received funding support from National Research Foundation of Korea and Green Cross Corporation, but the sponsors are neither involved in the study design nor its proposed operations. The study will be conducted in full accordance with the guidelines for Good Clinical Practice and the Declaration of Helsinki. The Korean Cancer Study Group Protocol Review Committee approved the study protocol (KCSG PC 22-11), and the approval of the Institutional Review Boards were obtained before the start of the patient recruitment at each institution (04-2022-021). Written informed consent will be obtained for all study participants by the principal investigator.

### 2.13. Dissemination

Our study results will be disseminated through presentations and publications. The results will be published as a journal article. All manuscripts will be authored by the study team and authorship will follow the established publication guidelines such as those of the International Committee of Medical Journal Editors.

## 3. Discussion

This study is a prospective randomized phase III study to verify the hypothesis that same-day administration of pegteograstim is non-inferior to next-day administration for 3 to 5 day chemotherapy regimens in which intensively myelosuppressive chemotherapy agents are administered on day 1. If the same-day method is non-inferior in regard to efficacy, patient convenience can be improved by removing the need for extension of hospitalization or revisit hospital to meet the next-day injection schedule.

The timing of administration of peg-GCSF is usually recommended to be 24 to 72 hours after completion of chemotherapy.^[1]^ If peg-GCSF is administered within 24 hours of completion of chemotherapy, myeloid progenitor cells are rapidly increased under stimulation of GCSF. The increased myeloid progenitor cells are depleted by myelosuppresive chemotherapeutic agents, thereby exacerbating CIN. This has been demonstrated in several clinical studies. In 2010, Burris et al^[3]^ showed that Gr4 ANC duration was shorter with the next-day administration (after 24 hours) than with the same-day administration (within 4 hours) method. The severity of CIN was also milder with the Next-day method. In addition, a large-scale retrospective cohort study showed that the same-day method was inferior to the next-day method for most evaluation factors.^[6]^

However, although the ASCO guidelines stipulate administration after 24 hours, these guidelines also recommend the same-day method for patients who have difficulties with next-day peg-GCSF administration.^[10]^ The reasoning behind this is that administration within 24 hours is better than not administering the peg-GCSF. Also, same-day administration showed improvement in Gr 4 CIN duration compared to next-day administration for ovarian cancers treated with the topotecan 5-day regimen. However, this was a subgroup analysis that did not reach statistical significance (same-day, 1.9 days; next-day, 2.4 days).^[3]^ In another retrospective study using 5-day DCF (Docetaxel, Cisplatin, and 5-FU) consisting of docetaxel and cisplatin on day 1, the same-day method showed superior outcomes in CIN incidence and FN prevention rates compared to the next-day method.^[8]^

We are now recruiting participants and the baseline database will be completed by 2023. Depending on the results, this study can officially promote the same-day administration of pegteograstim. This method has been unofficially applied on day 1 based on the results of retrospective studies or subgroup analyses of multi-day chemotherapy regimens that include administration of intensively myelosuppressive agents on the first day of chemotherapy.

## Acknowledgments

This research was sponsored by Green Cross Corporation.

## Author contributions

**Conceptualization:** Kwonoh Park.

**Data curation:** Kwonoh Park, Young-Kyung Jeon, Jung Hoon Kim, Younak Choi, Jae-Joon Kim, Sang-Bo Oh, So Yeon Oh, Seok Jae Huh, Ilhwan Kim, Seong Hoon Shin.

**Formal analysis:** Kwonoh Park.

**Funding acquisition:** Kwonoh Park.

**Investigation:** Kwonoh Park, Young-Kyung Jeon, Jung Hoon Kim, Younak Choi.

**Methodology:** Kwonoh Park, Sang-Bo Oh, Yun Jeong Hong.

**Project administration:** Kwonoh Park.

**Supervision:** Kwonoh Park.

**Writing – original draft:** Jae-Joon Kim.

**Writing – review & editing:** Yun Jeong Hong.
